# User QoS-Based Optimized Handover Algorithm for Wireless Networks

**DOI:** 10.3390/s23104877

**Published:** 2023-05-18

**Authors:** Hung-Chi Chu, Chia-En Wong, Wei-Min Cheng, Hong-Cheng Lai

**Affiliations:** 1 Department of Information and Communication Engineering, Chaoyang University of Technology, Taichung 413310, Taiwan; 2 Department of Computer Information and Network Engineering, Lunghwa University of Science and Technology, Taoyuan 333326, Taiwan

**Keywords:** handover, QoS, ping-pong effect

## Abstract

Due to the development of wireless network technology, various applications relying on good network quality are widely used on mobile devices. Taking the commonly used video streaming service as an example, a network with high throughput and low packet loss rate can meet the service requirements. When the moving distance of the mobile device is greater than the signal coverage of the AP, it will trigger the handover process to connect to another AP, and cause the network to disconnect and reconnect instantaneously. However, frequently triggering the handover procedure will cause a significant drop in network performance and affect the operation of application services. In order to solve this problem, this paper proposes the OHA and OHAQR. The OHA considers whether the signal quality is good or bad, and uses the corresponding HM method to solve the problem of frequent handover procedures. The OHAQR integrates the QoS requirements of the throughput and packet loss rate into the OHA with the Q-handover score, to provide high-performance handover services with QoS. Our experimental results show that the OHA and OHAQR have 13 and 15 handovers in a high-density scenario, respectively, and are better than the other two methods. The actual throughput and packet loss rate of the OHAQR are 123 Mbps and 5%, respectively, and the network performance is better than that of other methods. The proposed method shows excellent performance in ensuring the network QoS requirements and reducing the number of handover procedures.

## 1. Introduction

In recent years, technologies such as IEEE 802.11 wireless networks and cellular networks (such as 4G) have matured [1], and the application services using these technologies have also gradually increased. At the beginning of the development of wireless network technologies, the main focus of research and development was achieving high quality of service (QoS) and high connection stabilities between access points (APs) and user equipment (UE), and also achieving high packet delivery ratio (PDR) [2]. As these wireless network technologies mature, the stability of network connections and the transmission rate of data exchange increase, and more applications now use wireless networks as a medium for data exchange.

When using IEEE 802.11 wireless networks for transmission with high throughput requirements, once the UE moves to the boundary of the AP’s transmission range, the AP that originally provided the network service should hand over its job to a nearby AP, which can provide higher throughput to the UE; otherwise, the UE will keep gaining low throughput, which will affect the QoS of the UE. However, on the contrary, if the UE does not have high throughput requirements, and the handover decision is still prioritized to providing high-throughput network service between the AP and the UE, this may raise the chance of unnecessary handovers. Handover refers to the process of an AP handing over its job of network provision to another AP. It mainly occurs when the original serving AP can no longer provide a satisfactory QoS for the UE, and handing over the right of network service provision is required. If the handover judgment is not designed based on the users’ environment of application, the handover decision will be inaccurate and unable to ensure that the handover occurs at the correct time. When the UE wanders between multiple nearby APs, the signal strengths of these APs are similar, so if there is no good handover design, the handover between APs will become frequent, resulting in the occurrence of the ping-pong effect. Because of this phenomenon, we found that when designing the handover algorithm and its threshold, a more delicate design should be adopted to effectively prevent the ping-pong effect and further improve the QoS of the network.

In this paper, we propose the optimal handover algorithm (OHA) and the optimal handover algorithm with QoS requirements (OHAQR) to reduce the number of handover procedure triggers and ensure the quality of service in the network. In the OHA, to consider whether the signal quality is good or bad, different hysteresis margins (HMs) are used to solve the problem of frequent handover procedures. Different HMs are designed according to different signal qualities to help reduce unnecessary handover procedures being triggered. In the OHAQR, the handover score Q is included in the OHA algorithm to start the handover procedure earlier when the current network performance does not meet the QoS requirements. The handover score Q is a measurement indicator based on the QoS requirements, which includes throughput and packet loss rate (PLR) [3].

The rest of this paper is organized as follows: In Section 2, we detail and analyze the related work proposed by other authors, organize the pros and cons of their proposed methods, and further clarify the goal of our research; in Section 3, we clearly explain our proposed handover algorithm, which is optimized based on users’ QoS requirements; in Section 4, we present the experimental results simulated in different densities of topologies and compare the network performances of the proposed handover algorithm with the methods proposed by other authors, through the Mininet-WiFi simulator; and in the last section, we conclude this paper based on our proposed methods and summarize their performance breakthroughs, while also briefly outlining our future research.

## 2. Related Work

**Figure 1:**
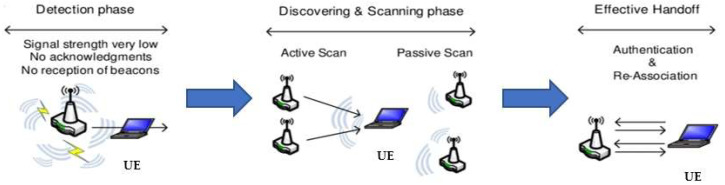
Wireless network handover procedure.

The handover procedure of wireless networks is an important issue. The processing flow of the handover procedure is shown in Figure 1. Before the handover process is executed, the system will enter the detection phase to start. In this process, the AP will detect the signal strength of the connection with the UE that it is currently connected to. If the signal quality of the connection between the UE and the AP is poor, the AP will notify the UE to start looking for nearby APs that it can hand over the right of network service provision to. After that, it will enter the second phase of the handover: the discovery and scanning phase. The UE will start to actively search for beacons sent by nearby APs, and further measure the signal strength of these connectable APs as a basis for the UE to select the best handover AP. The last stage is the effective handover. At this stage, after the UE selects the target AP for handover, it will verify and connect/reconnect with the AP. The handover procedure will be completed when all three of the above handover phases are completed.

Handover is divided into soft handover and hard handover [4]. In soft handover, the UE can still connect to the currently connected AP before the new connection with the target handover AP is established, which is usually performed via IEEE 802.11 wireless local area network (WLAN). In hard handover, which is used by 4G [5] mobile phone mobile communication technology, the UE must disconnect from the currently connected AP before the connection with the target handover AP can be established. In addition, handover can also be separated into vertical handover [6] and horizontal handover [4]. Vertical handover is also known as heterogeneous network handover; it refers to the behavior of the UE switching the connection between APs under two different network communication technologies. Horizontal handover is also known as homogeneous network handover. This handover method refers to when the UE switches between two APs with the same network communication technology. The goal of our research is to achieve optimized handovers between APs based on homogeneous networks.

In recent years, there have been many papers focusing on the design of handover algorithms. When designing handover methods, the handover threshold must be precisely designed, otherwise the connection between the UE and the AP will become unstable. There are three main handover decision methods: the received signal strength indicator (RSSI) threshold method, the dwell timer method, and the time prediction for staying method. The RSSI threshold method [7] is the most commonly used and the simplest goal for designing this kind of handover decision. It involves setting a threshold as the criterion for judging whether to perform the handover, which is called the “single-threshold handover judgment method” [8]. Although this method is easily applicable to various handover algorithms, it may cause frequent ping-pong effects and make wireless network connections unstable. The dwell timer method [9] adds a countdown timer on the basis of the “single-threshold handover judgment method”. When the signal strength between the nearby AP and the UE is stronger than the signal strength between the AP to which the UE is currently connected and the UE, the system will start a countdown timer (the countdown timer can be set by the user). When the countdown timer ends, it means that the signal fluctuation effect caused by an unstable signal between the UE and the nearby APs that can be handed over to is eliminated, meaning that the handover procedure can be executed. This method can reduce the probability of unnecessary handovers, but if the handover decisions of multiple connections are executed at the same time, multiple countdown timers will start at the same time, resulting in a high computational burden of the system. The time prediction for staying method [10] calculates the distance that the UE travels from the preset AP coverage boundary to the coverage boundary of the single handover threshold when the UE moves at a constant speed, to calculate the time that the UE travels within the coverage of the target handover AP. If the movement time of the UE calculated by the aforementioned method is higher than the right to delay time of the handover, the network service provision right will be transferred to the target handover AP. Although this method can reduce the generation of the ping-pong effect, like the dwell countdown timer method, the time estimation for staying needs to continue calculating, resulting in a high computational burden of the system. In addition to the design of a handover algorithm with a fixed threshold, the “dynamic threshold” approach was proposed [11]. In this approach, the available spectrum is allocated to the UE newly connected to the AP by setting dynamic thresholds. The “dynamic threshold” changes its value as the environment changes, so it can be considered the best solution in a changing environment. In this way, the flexibility of the system and its applicability to various environments will be further improved. Although this method should have good performance, it should be dynamically adjusted to the best handover parameters according to the environment; otherwise, it will still cause a ping-pong effect and reduce the stability of the user’s network service.

In order to alleviate the ping-pong effect, in [12], the “time prediction for staying method” was used, and whether the target handover AP could provide users with sufficient throughput was used as a handover decision indicator to reduce the overall number of handovers and improve network service efficiency. In [13], the author decided whether an AP could be selected as the target handover AP by checking whether the UE was facing it. After all nearby APs that can be handed over to are included in the target handover candidate list, one of the APs with the best signal strength between it and the UE will be selected as the target handover AP. At the same time, the signal strength between the selected target handover AP and the UE must be greater than the handover threshold to ensure that this AP can truly provide good network services to the UE.

Another way to deal with ping-pong effects is to use hysteresis margins (HMs) [14]. HMs are like a buffer space added to the handover threshold, so that the new handover threshold will increase the value of HMs to the original threshold. After adding this parameter to the handover algorithm, if the signal strength between the UE and the currently connected AP is lower than the original threshold, the handover that should have been performed before the HM was added will not be performed, because the signal strength between the UE and its currently connected AP is not bad enough. The handover procedure will only be executed after the handover condition is met. Therefore, when considering the long-term disconnection caused by the ping-pong effect, which results from the user’s wandering between two APs and further causing the UE to continuously switch service providers between APs, the use of HMs can greatly reduce the probability of the ping-pong effect and reduce network service interruption. In [14], a handover algorithm based on a double threshold and a double hysteresis margin was proposed. From the experimental results, although the method proposed by the author can achieve 100% PDR, more than double the expected number of handovers occurred in the experiment. It was found that the value of certain parameters in the handover algorithm, such as the handover threshold and HM, must be accurately designed; otherwise, this may cause unnecessary handovers and is not conducive to providing high-QoS network services.

In addition, in the handover process of a wireless network, it is also important to consider the factors of QoS to meet the needs of users. QoS [15] refers to the way that a network can use various basic technologies to provide better network service capabilities for specific network users, and is also a technology used to solve problems such as network delay and congestion. QoS is a control mechanism that provides different priority levels for different users or different data traffic, or according to the requirements of the application, to ensure that the performance of data traffic can reach a certain level. The measurement indicators of QoS include [16]: bandwidth, packet loss rate, error, delay, jitter (the delay difference may seriously affect the quality of streaming video and audio), and so on. QoS can also be prioritized for application data traffic to ensure that applications with high QoS requirements can receive enough QoS. In [17], a scoring criterion for the handover decision, Q-score, is proposed. The Q-score is obtained by multiplying the set network service-quality-related parameters (such as RSSI, bandwidth, etc.) by the individual weights. After that, the nearby AP with the highest Q-score will be selected as the UE target handover AP. However, in the handover algorithm proposed, there is a handover condition: if the application currently being used by the UE has a demand for real-time data transmission, the connection will immediately switch to the target handover AP. However, from another point of view, if the above conditions continue to be met, they will continue to hand over. As a result, the PDR of the overall network service will drop significantly. Therefore, according to the goal that we want to achieve, of reducing the number of handovers, the immediacy of the application will not be included in the judgment condition of the handover decision to reduce the number of disconnections. However, if the user has high QoS requirements, the system will try to achieve the QoS required by the user as much as possible, regardless of the cost caused by frequent handovers. In [18], an SDN-based RSSI handover mechanism and an SDN-based AP load-balancing handover algorithm are proposed. The former, as the station is associated with the AP providing the strongest signal, may result in reduced QoS when the AP is overloaded. The latter balances the load when the AP is in an overlapping area, thereby ensuring good QoS for all stations. From the experimental results, it can be seen that the AP load-balancing handover algorithm performed better in terms of transmission, bandwidth, jitter, and packet loss. In [19], a QoS-aware flexible mobility management scheme for software-defined networking (SDN)-based mobile networks is proposed. It classifies flows into four classes based on the QoS requirements of the service in terms of latency and loss tolerance. According to the priority of flow classification, a QoS-based differential handover process is provided for each class. Performance analysis shows that the proposed scheme can realize the flexible utilization of network resources and can effectively meet the QoS requirements of each class.

## 3. User QoS-Based Optimized Handover Algorithm

In this section, the optimized handover algorithm (OHA, also called Algorithm 1) and the optimized handover algorithm with QoS requirement (OHAQR, called Algorithm 2) are proposed. The former algorithm helps the UE to perform the minimum number of handovers and the latter prevents the AP from making the throughput and PLR lower than the user’s requirements. The details of these two handover algorithms are explained in the following subsections.

### 3.1. Optimized Handover Algorithm

An improved handover algorithm referenced in [14] was proposed to further help the system to select the best nearby handover AP and avoid unnecessary handovers. For the “single-threshold handover judgment method”, when the RSSI between the UE and the currently connected AP is inferior to the RSSI between the UE and the nearby connectable APs with the best RSSI between the UE, the connection with the currently connected AP will be transferred to the AP with the best RSSI with the UE among the nearby APs. The definition of RSSI is as follows [20]:

(1) $$RSSI=P_{r}-P_{L}\left(d_{0}\right)-10n\;log10\left(d_{i}/d_{0}\right)+X_{0}$$

$P_{r}$ represents the transmission power of the signal. $P_{L}\left(d_{0}\right)$ represents the path loss of the signal per unit distance $d_{0}$ due to environmental factors. $d_{i}$ is the distance between the actual position of the UE and the set reference point. $d_{0}$ is the mentioned unit distance. n is the attenuation factor of the signal. $X_{0}$ is a random value of Gaussian distribution.

**Figure 2:**
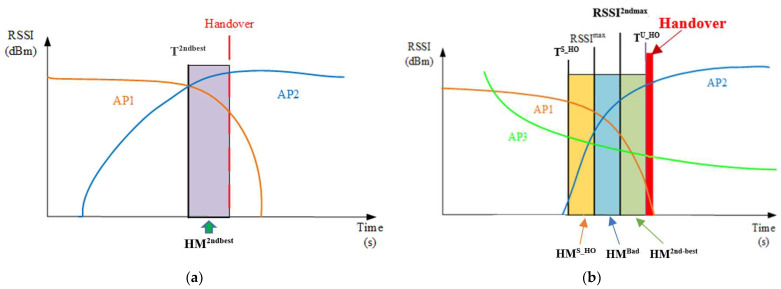
Optimized handover algorithm. (**a**) The overstep AP handover (taking AP1 and AP2 as an example); (**b**) the non-overstep AP handover (taking AP1, AP2, and AP3 as an example).

The algorithm of the OHA is described in Algorithm 1. When the system is started, the UE will start to search for nearby APs, start to collect the relevant information of these APs, and exclude the data of the AP that the UE is currently connected to from the collected data. Afterward, the UE will start to test the maximum throughput capability, and the severity of the packet loss problem between the server and itself will be the basis for further handover decisions. If the system finds that there are other APs nearby that can be handed over, it will start to check the current signal strength (*RSSI^C^*) of the connection between the UE and the AP. If the measured signal strength is greater than or equal to the threshold (*T*) set by the user, it means that it is in the “good signal condition range”, which means that its current connection signal quality is good and there is no urgent need to hand over (as shown in Figure 2a, and lines 3 to 7 of Algorithm 1). Note that the two curves in Figure 2a represent the RSSIs of AP1 and AP2, respectively. In this sense, the handover decision tends to reduce the probability of triggering the handover procedure. Conversely, if the measured signal strength is less than or equal to the threshold set by the user, this means that when it is in the “bad signal condition range”, we set an increased basis for decision-making for handover than in the good signal condition range, to prevent the UE from encountering poor connection or low service throughput (as shown in Figure 2a, and lines 9 to 13 of Algorithm 1).

Regardless of whether the current signal connection quality is in the range of good or bad signal conditions, the system will first make a rigorous “overstep AP handover” judgment to decide whether to directly hand over to the AP that can provide the second-best service quality. The method is that when we find the farthest AP in the direction that the UE is moving, the system will ensure that the UE can receive stable network services after connecting with it, to prevent the service instability problem caused by frequent handovers when the UE is handed over to the AP that can provide the best signal quality, and when it is about to leave the signal coverage of this AP, the UE then needs to switch the connection to another AP that can provide the best signal. Therefore, by judging the “signal strength of the second-best nearby AP” and the “UE heading direction”, to determine whether the user will move to the coverage of the nearby AP with the second-best service quality, and only when the following judgment conditions are met at the same time, will the handover procedure be triggered. First, when the signal strength of the second-best AP (*RSSI^2nd-best^*) is greater than the handover threshold of nearby AP with the second-best signal strength (*T^2nd-best^*) minus the handover HM of nearby AP with the second-best signal strength (*HM^2nd-best^*), it means that the nearby AP with the second-best signal strength can provide good enough signal quality. However, the HM used in this article is the upper/lower limit range of the tolerable signal strength variation based on a signal strength RSSI value, so the “subtract with HM” method is not a direct subtraction of the two signal strength levels. Second, when the UE moves towards the AP with the second-best service quality nearby, if the conditions for the overstep AP handover have not been established, which means that there is no AP that meets the second-best quality and can be handed over to, then the following more detailed and accurate handover judgment is performed to avoid unnecessary handovers.

This method divides the signal strength of the currently connected AP into “good signal quality” and “bad signal quality” to optimize the performance of the handover algorithm. When the current signal connection quality is within the good signal conditions, the following four conditions need to be satisfied, and the connection of the UE will be handed over to the AP with the best signal quality nearby.

1. The signal strength quality of the AP near the UE is the best (*RSSI^Max^*) and better than the sum of the signal strength of the currently connected AP (*RSSI^C^*) and the HM with a good signal quality (*HM^Good^*). Since the signal strength quality of neighboring APs is excellent, the handover procedure can be performed (i.e., *RSSI^Max^* ≥ *RSSI^C^* + *HM^Good^*).
2. “The minimum signal quality threshold for should handover” (*T^S_HO^*) is better than the sum of the signal strength of the currently connected AP (*RSSI^C^*) and “the minimum signal quality HM for should handover” (*HM^S_HO^*). The handover process is not performed until the signal strength quality of the currently connected AP is in this condition, which can effectively reduce the occurrence of the ping-pong effect (i.e., *T^S_HO^* ≥ *RSSI^C^* + *HM^S_HO^*).
3. The signal strength quality of the AP near the UE is the best (*RSSI^Max^*) and better than the sum of the signal strength of the second-best AP (*RSSI^2nd-best^*) and the HM with the second-best signal strength in the good signal interval (*HM^2ndgood^*). This means that for the UE, the quality of service of the nearby AP with the best signal strength is much better than the service quality of the second-best nearby AP (i.e., *RSSI^Max^* ≥ *RSSI^2nd-best^* + *HM^2ndgood^*).
4. When selecting the best nearby AP for handover, the AP with the largest signal increment will be selected as the best handover AP; that is, the UE keeps moving to it (i.e., UE is moving toward *RSSI^Max^* AP).

If the current signal connection quality is in the bad signal condition range (as shown in Figure 2b, and lines 9 to 13 of Algorithm 1), the handover procedure will be triggered when the following five conditions are met.

1. The signal strength quality of the AP near the UE is the best (*RSSI^Max^*) and better than the sum of the signal strength of the currently connected AP (*RSSI^C^*) and the HM with a bad signal interval (*HM^Bad^*). Since the signal strength quality of neighboring APs is excellent, the handover procedure can be performed (i.e., *RSSI^Max^* ≥ *RSSI^C^* + *HM^Bad^*).
2. The minimum signal quality threshold for handover (*T^S_HO^*) is better than the sum of the signal strength of the currently connected AP (*RSSI^C^*) and “the minimum signal quality HM for should handover” (*HM^S_HO^*). The handover process is not performed until the signal strength and quality of the currently connected AP are in this condition, which can effectively reduce the occurrence of the ping-pong effect (i.e., *T^S_HO^* ≥ *RSSI^C^* + *HM^S_HO^*).
3. The signal strength quality of the AP near the UE is the best (*RSSI^Max^*) and better than the sum of the signal strength of the second-best AP (*RSSI^2nd-best^*) and the HM with the second-best signal strength in the bad signal quality (*HM^2ndbad^*). This means that for the UE, the quality of service of the nearby AP with the best signal strength is much better than the service quality of the second-best nearby AP (i.e., *RSSI^Max^* ≥ *RSSI^2nd-best^* + *HM^2ndbad^*).
4. When selecting the best nearby AP for handover, the AP with the largest signal increment will be selected as the best handover AP; that is, the UE keeps moving to it (i.e., UE is moving toward *RSSI^Max^* AP).
5. “The worst acceptable signal strength that urgently requires handover” (*T^U_HO^*) should be considered as a top priority when making handover decisions. If the signal strength between the UE and the currently connected AP is lower than the worst acceptable signal strength that urgently requires handover for the connection, this means that the signal quality of the current connection is extremely poor, and the UE must immediately initiate a handover procedure to connect to a nearby AP with better signal quality (i.e., *T^U_HO^* ≥ *RSSI^C^*).

In other connection situations, such as when the UE has not been connected to any AP, the algorithm proposed in this paper will require the UE to check whether there is an AP nearby that can be connected to. If there is, it will connect to the one with the strongest signal strength between it and the UE; if not, it will wait until the UE moves to a nearby AP that can be connected to, and then connect to the one with the strongest signal between it and the UE. A schematic diagram of its operation is shown in Figure 2b. The advantage of the proposed algorithm is that it enables the UE to connect to the most stable and most suitable AP for its connection. Assume that the number of all APs is *n*. The proposed OHA algorithm needs to calculate the difference in signal strength between the AP that the UE is currently connected to and other APs in the remaining nearby range, and take into account *HM* and *T* as the basis for whether to perform the handover procedure. The calculation of the difference in signal strength between APs is at most *n* − 1 times, so it can be deduced that the computational complexity of this algorithm is *O*(*n*).
**Algorithm 1** Optimized Handover Algorithm1:  **Function** HAND-OFF TRIGGERING2:   **if** (*RSSI^C^* ≥ *T*) **then**3:     **if** (*RSSI^2nd-best^ ≥ T ^2nd-best^*−*HM^2nd-best^*) **AND** (UE is moving toward *RSSI^2nd-best^* AP) **then**4:        **return** *True*5:      **else if** (*RSSI^Max^* ≥ *RSSI^C^* + *HM^Good^*) **AND** (*T ^S_HO^* ≥ *RSSI^C^***+**
*HM^S_HO^*)        **AND** (*RSSI^Max^* ≥ *RSSI^2nd-best^* + *HM^2ndgood^*) **AND** (UE is moving toward *RSSI^Max^* AP) **then**6:        **return** *True*7:     **end if**8:   **else**9:     **if** (*RSSI^2nd-best^ ≥ T ^2nd-best^*−*HM^2nd-best^*) **AND** (UE is moving toward *RSSI^2nd-best^* AP) **then**10:       **return** *True*   11:     **else if** [(*RSSI^Max^* ≥ *RSSI^C^* + *HM^Bad^*) **AND** (*T ^S_HO^* ≥ *RSSI^C^* + *HM^S_HO^*)        **AND** (*RSSI^Max^* ≥ *RSSI^2nd-best^*+ *HM^2ndbad^*) **AND** (UE is moving toward *RSSI^Max^* AP)]        **OR** (*T ^U_HO^* ≥ *RSSI^C^*) **then**12:      **return** *True*13:    **end if**14:   **end if**15:   **return** *False*16: **end function**

### 3.2. Optimized Handover Algorithm with QoS Requirements (OHAQR)

The proposed handover algorithm based on the user’s high QoS requirements is shown in Algorithm 2; the algorithm will perform the best handover according to the settings of the user’s service throughput and PLR requirement, and the trade-off results obtained after individual weighting of decisions are used as indicators for evaluation. The QoS metrics mentioned above have high priority in handover decisions. Combined with the “optimized handover algorithm” proposed in this paper, if the QoS-based handover conditions are satisfied, even if the three handover conditions mentioned in the optimized handover algorithm that need to be satisfied at the same time are not met, the handover procedure will still be triggered. The trigger conditions for the handover are as follows.

**Figure 3:**
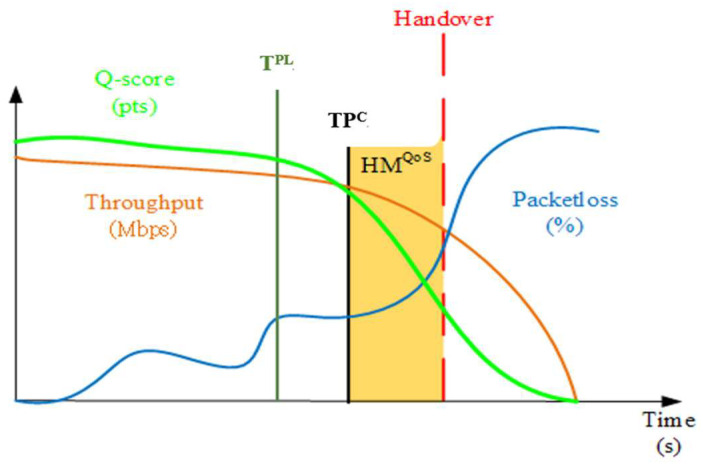
Schematic diagram of the operation of the optimized handover algorithm based on user QoS requirements.

If the user enables the setting of high QoS requirements, the weight of handover decision parameters with high QoS requirements can also be set according to the attributes and requirements of the application used by the user. Through a weight-based handover decision such as that in [16], we designed a trade-off strategy between throughput and PLR based on user settings for different application scenarios. In the case of maintaining high throughput, if the current throughput (*TP^C^*) capacity between the UE and the currently connected AP is lower than the “minimum throughput requirement threshold (*T^QoS^*)” minus the “minimum throughput requirement HM (*HM^QoS^*)”, this will immediately trigger the handover procedure (as shown in Figure 3, and line 11 in Algorithm 2). At the same time, if a PLR handover decision parameter with a lower weight is set, the handover algorithm will also consider the PLR performance of the service network. If the low PLR exceeds the tolerable level according to the set weight, although the throughput performance is still within an acceptable range, the low PLR performance will further affect the user’s network experience. At this time, the handover procedure is also triggered to immediately alleviate the low PLR problem, thereby improving the user’s QoS. On the contrary, if the PLR is a network performance indicator that users attach more importance to, if the PLR is lower than the threshold set by the user, it will immediately hand over, and if the weight of throughput of the handover decision has been set, the throughput performance will also be considered, and the best execution timing for handover is determined based on its weight to ensure that the user can always have sufficient QoS during the use of the network. The Q-score curve in Figure 3 is the QoS score variation of each data sampling point obtained after the trade-off calculation of Equation (2).

In addition to considering throughput and PLR, the immediacy of the application is also considered. If the AP currently being used by the UE requires a low-latency network environment, the system will consider the immediacy when making a handover decision to provide a low-latency network experience at all times. In addition, signal interference between APs was found after experiments and, although it did not seriously affect the network experience, we still used the number of nearby APs that could be searched by the UE as the number of interference signal sources. If there are many nearby interference sources, the overall QoS score will be lowered. Based on the above parameters, the values, in the range of 0~1, obtained by the normalization algorithm are multiplied by the individual weights of each parameter. After these values are added up, the result obtained is used to define the Q-handover score of handover decisions. When the Q-handover score is lower than the “user-defined QoS threshold (*T^QoS^*)”, the handover procedure will be triggered. The definition of the Q-handover score (*Q*) is as follows:

(2) $$Q=W_{TP}\times TP_{s}+W_{PL}\times PL_{s}+W_{AT}\times AT_{s}+W_{RSSI}\times RSSI_{s}$$

(3) $$TP_{s}=\frac{TP^{C}-TP^{Min}}{TP^{Max}-TP^{Min}}$$

(4) $$PL_{s}=1-\frac{PL^{C}-PL^{Min}}{PL^{Max}-PL^{Min}}\;$$

(5) $$AT_{s}=\frac{AT^{C}-AT^{Min}}{AT^{Max}-AT^{Min}}$$

(6) $$RSSI_{s}=1-\frac{RSSI^{C}-RSSI^{Min}}{RSSI^{Max}-RSSI^{Min}}$$

(7) $$W_{TP}+W_{PL}+W_{AT}+W_{RSSI}=1$$

where *W* represents the weight; *W_TP_*, *W_PL_*, *W_AT_*, and *W_RSSI_* represent the weights of throughput, packet loss rate, application type, and received signal strength gain, respectively; *TP* represents the throughput; and *TP^c^*, *TP^Min^*, and *TP^Max^* are the current, minimum, and maximum throughputs, respectively. Throughput is set between 0 and 150 Mbps. *PL* represents the packet loss rate, and *PL^c^*, *PL^Min^*, and *PL^Max^* are the current, minimum, and maximum packet loss rates, respectively. The packet loss rate can be set from 0 to 100%. *AT* represents the application types, and *AT^c^*, *AT^Min^*, and *AT^Max^* are the current, minimum, and maximum application types, respectively. The application types can be set from 1 to 10. The application type is used to indicate the priority level of the application service, and its value is 1~10: 1 indicates the lowest priority and 10 indicates the highest priority. *RSSI* represents the received signal strength indicator, and *RSSI^c^*, *RSSI^Min^*, and *RSSI^Max^* are the current, minimum, and maximum interference received signal strength indicators, respectively. The received signal strength indicator is expressed as the sum of all surrounding receivable signal strengths. Suppose *RSSI^Min^* is −90 dBm and *RSSI^Max^* is −1 dBm.

The proposed OHAQR is to add a QoS evaluation mechanism in the OHA to measure whether to perform the handover procedure. Since the calculation complexity of OHA is *O*(*n*), and the evaluation and judgment of QoS includes the QoS calculation of at most *n* APs, the calculation complexity is *O*(*n*). Since QoS calculation is required for each handover judgment, the overall computational complexity of the OHAQR algorithm is *O*(*n^2^*).
**Algorithm 2** Optimized Handover Algorithm with QoS Requirements1:  **Function** HAND-OFF TRIGGERING2:   **if** (*RSSI^C^* ≥ *T*) **then**3:     **if** (*RSSI^2nd-best^ ≥ T ^2nd-best^*−*HM^2nd-best^*) **AND** (UE is moving toward *RSSI^2nd-best^* AP) **then**4:        **return** *True*5:      **else if** (*RSSI^Max^* ≥ *RSSI^C^* + *HM^Good^*) **AND** (*T ^S_HO^* ≥ *RSSI^C^*
**+**
*HM^S_HO^*)        **AND** (*RSSI^Max^* ≥ *RSSI^2nd-best^* + *HM^2ndgood^*) **AND** (UE is moving toward *RSSI^Max^* AP) **then**6:        **return** *True*7:     **end if**8:   **else**9:     **if** (*RSSI^2nd-best^ ≥ T ^2nd-best^*−*HM^2nd-best^*) **AND** (UE is moving toward *RSSI^2nd-best^* AP) **then**10:       **return** *True*   11:     **else if** [(*RSSI^Max^* ≥ *RSSI^C^* + *HM^Bad^*) **AND** (*T ^S_HO^* ≥ *RSSI^C^*
**+**
*HM^S_HO^*)        **AND** (*RSSI^Max^* ≥ *RSSI^2nd-best^* + *HM^2ndbad^*) **AND** (UE is moving toward *RSSI^Max^* AP)]        **OR** (*Q* < *T^QoS^*)       **OR** (*T ^U_HO^* ≥ *RSSI^C^*) **then**12:      **return** *True*13:    **end if**14:   **end if**15:   **return** *False*16: **end function**

## 4. Experimental Results

The simulation experiments were performed using Mininet-WiFi [21,22], which is an open-source platform for simulating wireless scenarios, allowing high-fidelity experiments that replicate real network environments. Mininet-WiFi is a wireless network emulator extended from Mininet. It has native WiFi support, but can also emulate other wireless network technologies in experiments using it. Users can virtualize stations and access points, as well as use existing Mininet nodes, such as hosts and switches. The hardware used in the simulation experiment was a notebook, which was a Windows 10 laptop with a 15.60-inch display that had a resolution of 1366 × 768 pixels. It was powered by a Core i5-8250U processor and came with 4 GB of DDR4 RAM. The notebook had 1 TB of hard disk storage. The graphics were powered by NVIDIA GeForce MX150.

The experimental scenarios and parameters will be described in Section 4.1. In Section 4.2, the performance and QoS comparisons between the proposed method and the other two hand-off methods will be shown, to demonstrate the excellent performance of the proposed method.

### 4.1. Experimental Scenarios

Since the experimental environment focuses on the design of the handover algorithm and its experimental performance analysis, on the issue of security we will assume that the wireless network environment of this study is a secure environment. APs were properly secured throughout the experiment to avoid unauthorized access or malicious attacks. This experiment considered three experimental scenarios of high-density wireless network deployment, low-density wireless network deployment, and sparse wireless network deployment.

**Figure 4:**
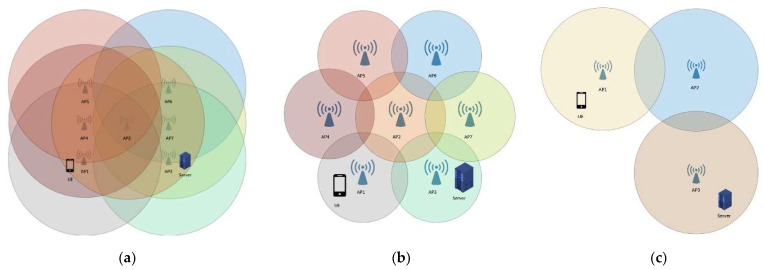
Experimental scenarios. (**a**) High-density wireless network deployment; (**b**) low-density wireless network deployment; (**c**) sparse wireless network deployment.

Scenario 1: High-density wireless network deployment. As shown in Figure 4a, a certain number of APs were deployed to cover a geographical area and any location in the area could receive at least two wireless network signals. This scenario was based on a large-scale environment, and the AP signal coverage distance was 125 m. An AP was placed on each of the two endpoints and the midpoint of a horizontal straight line with a length of 50 m (i.e., AP4, AP2, AP7, and the distance between adjacent APs was 25 m). Above the two endpoints perpendicular to the straight line, an AP was placed 25 m below each (that is, AP1, AP3, AP5, AP6, and the distance between adjacent APs was 25 m). The UE could receive signals from at least four APs at the same time and could receive signals from up to seven APs at the same time. In addition, a server could provide the data required by the UE. The signal coverage range of AP1~AP7 is the circle that covers each AP in the figure, with each color corresponding to a different AP.

Scenario 2: Low-density wireless network deployment, as shown in Figure 4b, deployed the minimum number of APs to cover a geographical area. This scenario was based on a large-scale environment, and the AP signal coverage distance was 125 m. One AP was placed at the center of the regular hexagon and six APs were placed at the six vertices of the regular hexagon. The distance between an AP and the adjacent AP was 100 m. The UE could receive signals from at least one AP at the same time and could receive signals from up to seven APs at the same time. In addition, a server could provide the data required by the UE. The signal coverage of AP1~AP7 is the circle that covers each AP in the figure, with each color corresponding to a different AP.

Scenario 3: Sparse wireless network deployment, as shown in Figure 4c, deploys APs only where there is a demand for wireless networks. This scenario was based on a small-scale environment, and the AP signal coverage distance was 25 m. AP1 and AP2 were placed horizontally at a distance of 40 m, and AP3 and AP2 were placed vertically at a distance of 30 m. The UE could receive signals from at least one AP at the same time and could receive signals from up to two APs at the same time. In addition, a server could provide the data required by the UE. The ranges of signal coverage of AP1~AP3 are the circles that cover each AP in the figure, with each color corresponding to a different AP.

The parameters of the experimental environments are shown in Table 1 and the parameters of the proposed handover algorithm are shown in Table 2. Note that in spare wireless network deployment, the signal coverage of the AP and the UE were both small, its *HM^Good^* was set as 5 dBm, and the *HM^Bad^* was set as 3 dBm [14].

### 4.2. Performance Analysis

In the above three scenarios, we used the built-in handover algorithm of Mininet-WiFi, the dual HM handover algorithm used by DoTHa [14], and the proposed OHA and OHAQR methods to compare the performance of the system. The experimental results included number of handovers, handover processing time, network transmission performance, and average throughput. Furthermore, to further demonstrate our methods, time-stamp-based statistics were collected from the experiments on the aforementioned switching algorithms, and we employed results performed in high-density topologies to clearly indicate the statistical distribution during the experiments. In Scenario 1, the UE moved from the position of AP1 to the position of AP2 in a straight line at a walking speed, and then moved from the position of AP2 to the position of AP3 in a straight line at a walking speed. In Scenarios 2 and 3, the UE started to move within the coverage of the seven APs and moved in the direction randomly calculated by the “random direction” mobility model. In the mobility model, the connection mechanism we applied was strongest-signal-first (SSF), because it more actively switched the UE’s connection to the AP that provides its best connection to prevent the UE from continuously connecting to the least-loaded APs with poor signal quality [23]. The iperf network traffic generation tool [24] was used as a test tool for network transmission performance, and the type of data transmitted was user datagram protocol (UDP) [25] packets. The test period of a single transmission was 1 s, and the maximum throughput transmitted during every period was 150 Mbps.

**Figure 5:**
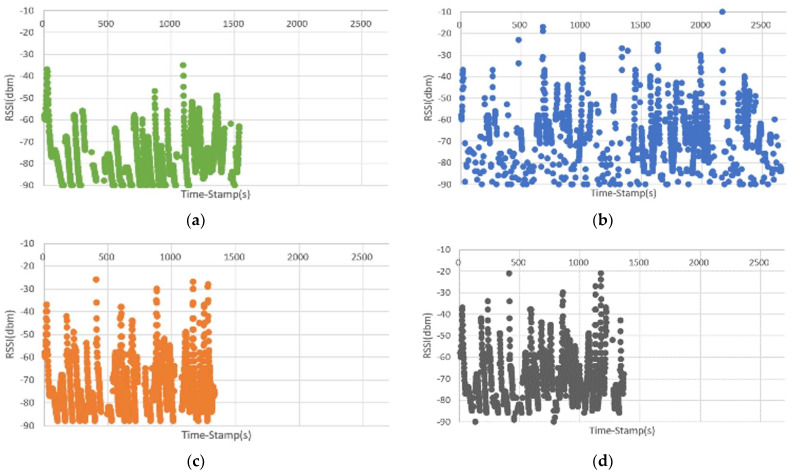
The relationship between the current RSSI and time of the UE using different handover algorithms: (**a**) Mininet-WiFi; (**b**) DoTHa; (**c**) OHA; (**d**) OHAQR.

In Scenario 1, four handover algorithms, Mininet-WiFi, DoTHa, OHA, and OHAQR, were used to observe the RSSI variation in UE during the experiment, as shown in Figure 5. Using the built-in handover algorithm of Mininet-WiFi in Scenario 1, it can be found in Figure 5a that the first connection was established at 8 s after the start of the experiment. During this period, the RSSI of the connection between the UE and the AP remained very stable because it was caused by connecting to the AP with the strongest RSSI. However, in Figure 5b, the handover algorithm of DoTHa was used. During the entire experiment period, a large number of cases of disconnection and reconnection occurred after the AP was disconnected. This is because in a high-density network environment, the signals affect each other, and the connected AP is frequently changed (causing the RSSI of the UE to be intermittent), resulting in the occurrence of the ping-pong effect. As shown in Figure 5c,d, the OHA and OHAQR algorithms proposed in this study made the RSSI of the UE remain stable most of the time.

**Figure 6:**
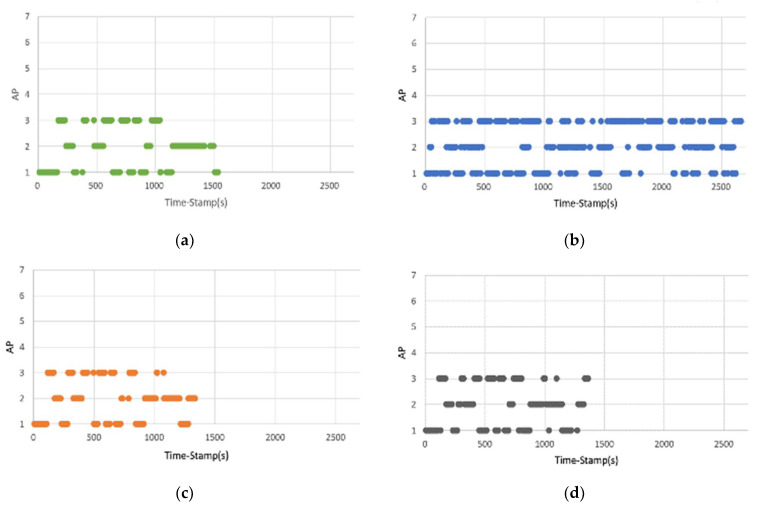
The relationship between the currently connected AP and time of the UE using different handover algorithms: (**a**) Mininet-WiFi; (**b**) DoTHa; (**c**) OHA; (**d**) OHAQR.

In Scenario 1, considering the time when the UE is connected to a specific AP, the experimental results show that using the built-in handover algorithm of Mininet-WiFi stabilized the connection with a specific AP, resulting in 17 handovers (see Figure 6a). However, using DoTHa’s handover algorithm generated a large number of handover situations, resulting in 248 handovers (see Figure 6b). This result shows that this method can cause connection instability problems. The handover algorithms using OHA and OHAQR also provided a stable connection with a specific AP, resulting in 13 and 15 handovers, respectively (see Figure 6c,d).

**Figure 7:**
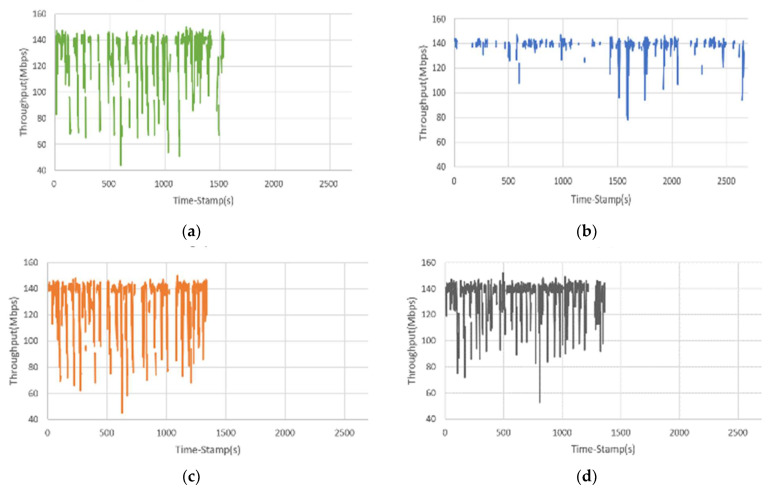
The relationship between the actual throughput and time of the UE using different handover algorithms: (**a**) Mininet-WiFi; (**b**) DoTHa; (**c**) OHA; (**d**) OHAQR.

Throughput is the rate at which a UE successfully sends or receives messages. Note that “actual throughput” refers to the amount of messages transmitted per second during the entire experiment period, including the time of disconnection or inability to transmit. The “average throughput” refers to the amount of messages transmitted per second during the entire experiment period, excluding the time of disconnection or inability to transmit. In Scenario 1, the experimental results considering the actual throughput and the average throughput of the UE show that the actual throughput and the average throughput using the built-in handover algorithm of Mininet-WiFi were 81 Mbps and 127 Mbps, respectively (see Figure 7a). The actual throughput and the average throughput of the handover algorithm using DoTHa were 106 Mbps and 134 Mbps, respectively (see Figure 7b). The actual throughputs and the average throughputs of the handover algorithms using OHA and OHAQR were 106 Mbps, 131 Mbps, 123 Mbps, and 135 Mbps, respectively (see Figure 7c,d). The results show that when the UE was connected to the AP, the average throughput of various handover algorithms could maintain more than 100 Mbps, but the actual throughput of the built-in handover algorithm of Mininet-WiFi was only 81 Mbps.

The experimental results on the average packet loss rate show that the average packet loss rate of the built-in handover algorithm using Mininet-WiFi was 8%, which was the worst among the four handover methods. The average packet loss rates of handover algorithms using DoTHa, OHA, and OHAQR were 5%, 7%, and 5%, respectively.

In Scenario 2 and Scenario 3, four handover algorithms were also used, and experiments were conducted on the number of handovers, average throughput, average packet loss rate, and actual throughput. The experimental results of the four handover algorithms in the three scenarios are summarized in Table 3.

In Scenario 1, the Avg. TP of the four algorithms was higher than 127 Mbps, and the PLR was lower than 8%. However, the number of handovers of the DoTHa algorithm was 248, which shows that its ping-pong effect was serious, and it was not suitable for this scenario. The proposed OHA algorithm was excellent in terms of the number of handovers and only handed over 13 times, which was 23.53% better than the Mininet-WiFi algorithm. The OHAQR algorithm required 15 handovers to ensure QoS requirements, but its Act. TP was 123 Mbps, Avg. TP was 135 Mbps, and PLR was 5%. The OHAQR algorithm had excellent performance and showed improvements over the Mininet-WiFi algorithm of 11.76%, 51.85%, 6.3%, and 37.5% in the number of handovers, Act. TP, Avg. TP, and PLR, respectively.

In Scenario 2, the Avg. TP of the four algorithms was higher than 125 Mbps, and the PLR was lower than 10%. However, the number of handovers of the DoTHa algorithm was still as many as 214, which shows that its ping-pong effect was serious. Although the Mininet-WiFi algorithm had the minimum number of handovers of 10 in this scenario, its Act. TP was 100 Mbps, Avg. TP was 125 Mbps, and PLR was 10%, which was worse than the other three algorithms. The number of handovers for the OHA algorithm was 17. The performance of the OHA algorithm was 119 Mbps for Act. TP and 133 Mbps for Avg. TP, which were 19% and 6.4% better than the Mininet-WiFi algorithm. In order to ensure the QoS requirements of the OHAQR algorithm, the number of handovers was 27. The performance of the OHAQR algorithm was 102 Mbps for Act. TP, 135 Mbps for Avg. TP, and 4% for PLR, which were 2%, 8%, and 60% better than the Mininet-WiFi algorithm.

In Scenario 3, the number of handovers of the four methods was less than or equal to two. The Mininet-WiFi algorithm had the smallest number of handovers, but it had the worst performance in Act.TP, Avg.TP, and PLR. The DoTHa algorithm had the best performance in avg. TP and PLR, showing that this is the most applicable scenario. The number of handovers of the OHA algorithm was two, its performance in Act. TP was 60 Mbps, and PLR was 12%, which were 33.33% and 7.69% better than the Mininet-WiFi algorithm, respectively. In order to ensure the QoS requirements, the OHAQR algorithm had two handovers, but its performance was 68 Mbps for Act. TP, 26 Mbps for Avg. TP, and 9% for PLR, which were better than the Mininet-WiFi algorithm by 51.11%, 5.88%, and 30.77%, respectively.

Based on the Mininet-WiFi algorithm, when the signal strength between the UE and the currently connected AP is lower than the minimum signal strength, the UE will automatically disconnect the connected AP. Then, the UE starts to search for nearby APs and performs a reconnection process to complete the handover process. This method will increase the number of handovers as the deployment density of APs in the scenario increases, resulting in poor performance in terms of throughput and packet loss rate.

The DoTHa handover algorithm only considers a single threshold *T* added to an *HM^Good^* or *HM^Bad^* to prevent unnecessary handovers from being triggered. The design of DoTHa handover algorithm is simple and only suitable for sparse wireless network topology. As long as the signal strength of the AP near the UE is greater than or equal to the sum of the signal of the AP currently connected to the UE and the HM, the handover process will be triggered. Since the algorithm mainly considers the handover algorithm designed in the sparse wireless network environment, it had better performance in Scenario 3. However, since the UE could receive a large number of APs with high signal strength in Scenarios 1 and 2, there were frequent handovers.

The proposed OHA considers whether the signal is good or bad and its judgment considers various conditions such as *HM^Good^*, *HM^Bad^*, *T^S_HO^*, *T^U_HO^*, etc., and so the performance of the number of handovers in Scenario 1 was the best. In Scenarios 2 and 3, because APs were deployed with a low density or sparsely, sometimes the UE could not obtain suitable candidate APs, resulting in a slight decrease in performance. OHAQR is an integrated algorithm based on the OHA algorithm and considering QoS requirements. Therefore, in order to meet the requirements of QoS, the performance of throughput and PLR will be maintained, and the number of handovers will increase slightly.

## 5. Conclusions

This paper proposes an optimized handover algorithm and an optimized handover algorithm with QoS requirements to improve the handover performance. The experimental results show that, in Scenario 1, the OHA algorithm and the OHAQR algorithm outperformed the Mininet-WiFi algorithm by 23.53% and 11.76%, respectively, in the number of handovers. The OHAQR algorithm had excellent performance and outperformed the Mininet-WiFi algorithm by 11.76%, 51.85%, 6.3%, and 37.5% in the number of handovers, Act. TP, Avg. TP, and PLR, respectively. Similarly, in Scenarios 2 and 3, the OHA algorithm and OHAQR algorithm were better than the Mininet-WiFi algorithm in terms of Act. TP, Avg. TP, and PLR. The DoTHa algorithm only had better Avg. TP and PLR in Scenario 3, but as there were more than 200 handovers in Scenarios 1 and 2, the ping-pong effect was serious. Since the OHAQR has to meet the requirements of QoS, the number of handovers was not the least, but it did have excellent performance in Act. TP, Avg. TP, and PLR.

In future work, we will continue to explore not only improving the system performance of the handover algorithm with QoS requirements, but also continue to explore the applicability of other network service quality indicators and handover algorithms. We expect to design a general handover algorithm suitable for various network service usage scenarios to provide an efficient and stable network usage environment.

## Figures and Tables

**Table 1:** The settings of the simulation environment parameters.

Parameter	Scenario 1	Scenario 2	Scenario 3
Number of APs	7	7	3
AP Coverage	125 m	125 m	25 m
AP Operating Mode	802.11 ac	802.11 ac	802.11 ac
Channel Testbed	5G Hz	5G Hz	5G Hz
Testruns	1000	1000	1000
UE Mobility Model	Random Direction	Random Direction	Random Direction
UE Moving Velocity	0.9~1.5 m/s	0.9~1.5 m/s	0.9~1.5 m/s

**Table 2:** The parameter settings of the proposed algorithm.

Symbol	Value	Symbol	Value
*T*	−70 dBm	*HM^2ndgood^*	40 dBm
*HM^Good^*	50 dBm	*HM^2ndbad^*	20 dBm
*HM^Bad^*	30 dBm	*T^QoS^*	0.78
*T^S_HO^*	−70 dBm	*HM^QoS^*	10
*HM^S_HO^*	5 dBm	*RSSI^Max^*	−1 dBm
*T^U_HO^*	−88 dBm	*RSSI^Min^*	−90 dBm

**Table 3:** Summary of the experimental results of the four handover algorithms in three scenarios.

Handover Algorithm Experimental Results		Mininet-WiFi	DoTHa	OHA	OHAQR
Scenario 1	Number of Handovers	17	248	13	15
	Act. TP (Mbps)	81	106	106	123
	Avg. TP (Mbps)	127	134	131	135
	Avg. PLR (%)	8	5	7	5
Scenario 2	Number of Handovers	10	214	17	27
	Act. TP (Mbps)	100	101	119	102
	Avg. TP (Mbps)	125	129	133	135
	Avg. PLR (%)	10	7	6	4
Scenario 3	Number of Handovers	1	2	2	2
	Act. TP (Mbps)	45	59	60	68
	Avg. TP (Mbps)	119	132	119	126
	Avg. PLR (%)	13	5	12	9

Note that Act. TP means actual throughput, Avg. TP means average throughput, and Avg. PLR means average packet loss rate.

## Data Availability

The datasets used in the current study are available from the corresponding author upon request.
